# Astrocyte Reprogramming in Stroke: Opportunities and Challenges

**DOI:** 10.3389/fnagi.2022.885707

**Published:** 2022-05-19

**Authors:** Zhouzhou Peng, Hui Lu, Qingwu Yang, Qi Xie

**Affiliations:** ^1^Department of Neurology, Second Affiliated Hospital, Army Medical University (Third Military Medical University), Chongqing, China; ^2^Chongqing Institute for Brain and Intelligence, Guangyang Bay Laboratory, Chongqing, China

**Keywords:** stroke, astrocyte reprogramming, transcription factors, lineage tracing, single-cell sequencing

## Abstract

Stroke is a major cause of morbidity and mortality worldwide. In the early stages of stroke, irreversible damage to neurons leads to high mortality and disability rates in patients. However, there are still no effective prevention and treatment measures for the resulting massive neuronal death in clinical practice. Astrocyte reprogramming has recently attracted much attention as an avenue for increasing neurons in mice after cerebral ischemia. However, the field of astrocyte reprogramming has recently been mired in controversy due to reports questioning whether newborn neurons are derived from astrocyte transformation. To better understand the process and controversies of astrocyte reprogramming, this review introduces the method of astrocyte reprogramming and its application in stroke. By targeting key transcription factors or microRNAs, astrocytes in the mouse brain could be reprogrammed into functional neurons. Additionally, we summarize some of the current controversies over the lack of cell lineage tracing and single-cell sequencing experiments to provide evidence of gene expression profile changes throughout the process of astrocyte reprogramming. Finally, we present recent advances in cell lineage tracing and single-cell sequencing, suggesting that it is possible to characterize the entire process of astrocyte reprogramming by combining these techniques.

## Introduction

Stroke is a disease associated with high morbidity, mortality, and disability, resulting in a heavy social and economic burden worldwide. Reports estimate that there were 12 million incident cases of stroke and 6 million deaths from stroke in 2019. Globally, stroke remains the second-leading cause of death and the third-leading cause of death and disability combined (GBD 2019 Stroke Collaborators, 2021).

Stroke occurs when blood flow to the brain is suddenly interrupted, usually because a blood clot dislodges from elsewhere in the body and becomes stuck in the arteries that supply the brain with fresh, oxygenated blood. When the artery becomes blocked by an embolus and cannot deliver oxygen to the body, brain cells die immediately. Notably, the massive death of neurons has serious consequences for the body after stroke. Previous literature has reported that in patients experiencing a typical large vessel acute ischemic stroke, approximately 120 billion neurons die every hour, causing irreversible damage (Saver, 2006). Recently, the recanalization rate after stroke has increased significantly with the popularity of mechanical thrombectomy and pharmacological thrombolysis (Menon et al., 2018; Yang et al., 2020), but there is currently no effective treatment to rescue such dying neurons.

Given the lack of evidence regarding the direct regeneration of neurons in adults, researchers have focused on the possibility of reprogramming other cells into neurons, particularly using stem cell-based regenerative therapies to rescue the central nervous system (CNS) following nerve injuries. The relevant literature reports that mesenchymal stem cells can be induced into neurons *in vitro*, but are poorly converted into neurons in host brain tissue due to xenograft rejection and interference from other cells in the brain (Park et al., 2020). Therefore, the use of stem cell regeneration to replenish post-stroke neurons remains controversial in humans.

## Astrocyte Reprogramming as an Alternative Therapy for Stroke

Astrocytes, the most numerous type of neuroglia in the brain, can be subdivided into protoplasmic and fibrous types. Protoplasmic astrocytes possess highly branched bushy processes and are widely distributed in the gray matter. They extend endfeet to blood vessels and enwrap them to form the glial limiting membrane, which is the outermost wall of the blood–brain barrier (BBB). However, fibrous astrocytes possess straight and long processes and are mainly located in white matter. In fibrous astrocytes, the expression level of glial fibrillary acidic protein (GFAP) is higher than that of protoplasmic astrocytes (Tabata, 2015).

Astrocytes play an important role in the normal physiological activities of the brain, which involve providing energy to neurons, removing metabolic waste, the process of neurovascular coupling, synaptogenesis, maintenance of the redox state, pH, and K^+^ balance, and neurotransmitter homeostasis (Verkhratsky and Nedergaard, 2018). Activated astrocytes play a dual role after stroke. They proliferate rapidly in the acute phase after ischemia, preventing further damage to the brain from various harmful substances, which provides a certain degree of protection in the pathogenesis of stroke. Simultaneously, the formation of a glial scar in the later stage of stroke interferes with the repair of brain tissue and the process of neurodegenerative (Liu and Chopp, 2016).

In addition to the above functions in the healthy CNS, astrocytes exhibit a strong response to CNS injury and disease, commonly referred to as reactive astrocytes, which are regarded as astrocytes undergoing morphological, molecular, and functional remodeling to exhibit high plasticity (Escartin et al., 2021). Furthermore, since astrocytes and neurons originate from the same precursor cells, recent studies have found that astrocytes have the potential to function in nerve regeneration and can be reprogrammed into neurons under specific conditions (Masserdotti et al., 2016). For example, previous studies have reported that by adjusting the expression of some specific transcription factors including NeuroD1, Mash1, Ascl1 etc. *in vivo* can convert endogenous glial cells directly into functional neurons (Ge et al., 2020). Thus, astrocytes are ideal candidates for reprogramming into neurons, which may act as an alternative therapy in the future for the replenishment of neurons after stroke.

## Astrocyte Reprogramming Methods

The current views on the reprogramming of astrocytes into neurons mainly include dedifferentiation and trans-differentiation. Dedifferentiation implies that the differentiated cells lose phenotypic characteristics under certain conditions and are reversed into stem cells, which can further proliferate and differentiate into mature cells (Koen and Rugg, 2019). Astrocytes can be reversed to form neural spheres, which have the characteristics of neural stem cells (NSCs) and possess self-renewal ability and multi-directional differentiation potential, and thus can differentiate into neurons, astrocytes, and oligodendrocytes *in vitro* (Huang and Tan, 2015). However, trans-differentiation, also known as lineage reprogramming, is the process by which a germ-derived cell or a pluripotent stem cell is transformed into another adult cell or pluripotent stem cell. Trans-differentiation usually occurs between similar tissues, which are adjacent to each other at the initial stage of embryogenesis. Often, the differential expression of one or more transcription factors determines their eventual different development directions (Amamoto and Arlotta, 2014; Wang H. et al., 2021).

The trans-differentiation of astrocytes can be further divided into direct trans-differentiation (DT) and indirect lineage reprogramming (ILC). DT means that astrocytes can be transformed directly into neurons by transcription factors (TFs), microRNAs, and some small molecules that regulate key developmental pathways, while ILC is a process that induces astrocytes to regress to a plasticized intermediate state. Such early intermediate cells can expand to form neuroblasts and then convert into neurons under certain conditions (Xu et al., 2015; Magnusson and Frisen, 2016). Unlike dedifferentiation, the entire process of ILC does not require the establishment of a pluripotent state (Jopling et al., 2011). Next, we elaborate on the process of astrocyte reprogramming from the aspects of dedifferentiation and trans-differentiation.

## Astrocyte Reprogramming Through Dedifferentiation

The 2012 Nobel Prize was awarded to John Gurdon and Shinya Yamanaka for demonstrating that the identity of differentiated cells is decided not because they are in an irreversible state, but because the population of these cells can retreat to a pluripotent state under appropriate environmental signals. Takahashi and Yamanaka (2006) identified four factors (Sox2, Klf4, Oct3/4, and cMyc) that could induce pluripotent stem cells (iPSCs) in mouse fetal and adult fibroblasts. This research has promoted many subsequent studies to demonstrate that various adult cells can be reversed into stem cell-like cells, which has also created a new research direction for the field of tissue repair and regeneration after CNS injury and degenerative diseases.

Additionally, these iPSCs can be further induced to generate neural progenitor cells expressing Nestin and Sox2, which can be induced to differentiate to mature neurons and astrocytes, respectively, (Nakajima-Koyama et al., 2015). Inversely, Ruiz et al. (2010) first transfected human cerebellar astrocytes with retroviruses that encoded Klf4, Sox-2, Oct-4, and c-Myc to convert them into hiPSCs *in vitro*. Then, Corti et al. (2012) demonstrated that progenitor and mature cells with overexpression of the single reprogramming factors OCT4, SOX2, or NANOG could be obtained by inducing reprogramming of human cortical astrocytes into neural stem/progenitor cells. Furthermore, Su et al. (2014) and Wang et al. (2016) first overexpressed Sox2 in astrocytes in a spinal cord injury model to reverse them into neuronal progenitor cells, and then induced them using small molecule compounds such as VPA, BDNF, and neurotrophic factors, or by knocking down the expression of p53 to induce differentiation into mature neurons. These studies have confirmed that astrocytes can be reversed into neural stem progenitor cells and then differentiated into neurons according to the iPSCs induction method and induction factor.

In addition to the factors identified in the Takahashi and Yamanaka (2006) study described above (Oct3/4, Sox2, Klf4, c-Myc), recent research suggests that when Notch signaling is blocked, astrocytes can transform into an NSC-like state (Zamboni et al., 2020). Although the current research-based on astrocyte dedifferentiation has achieved good results, many precise regulatory steps are urgently required to induce the differentiation of dedifferentiated NSCs into functional neurons, which has not yet been fully clarified.

## Astrocyte Reprogramming Through Trans-Differentiation

**FIGURE 1:**
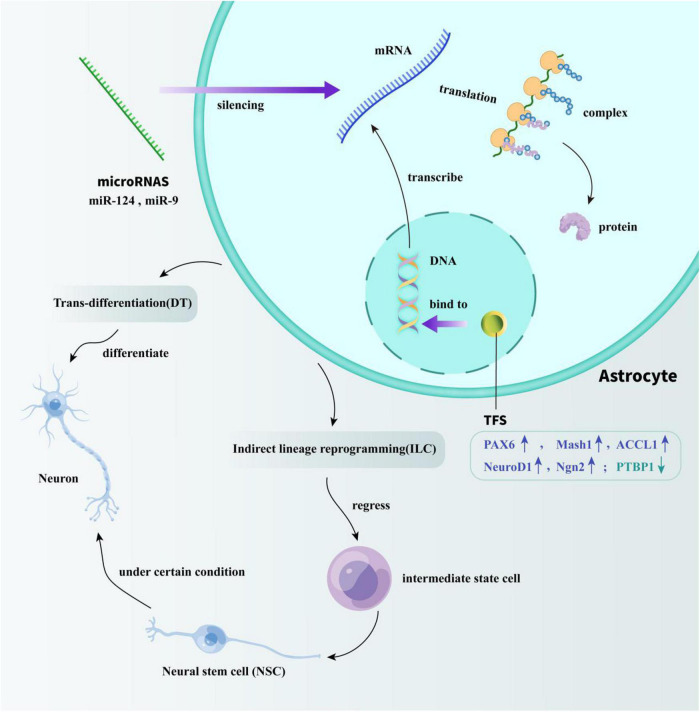
Specific mechanisms of TFs and microRNAs acting on astrocyte reprogramming. TFs modulate the efficiency of gene transcription by binding to DNA-regulatory sequences. miRNAs play a regulatory role by silencing transcriptionally generated mRNA. Through the regulation of TFs and miRNA, the trans-differentiation of astrocytes into neurons *in vivo* can be successfully achieved. One way is directly reprogramming astrocytes into neurons. The other way is by regressing astrocytes into intermediate state cells, and then differentiate into NSCs to indirectly obtain mature neurons.

The trans-differentiation of astrocytes can be divided into two types: DT and ILC. Compared to ILC, the research on the process of DT has attracted more attention, which can occur both *in vivo and in vitro.* According to previous studies, the DT methods of astrocytes include the use of transcription factors (TFs), microRNAs, and some small molecules, all of which are introduced in the next section and shown in Figure 1 in the form of cartoon picture.

### Astrocyte Reprogramming Through Transcription Factors

TFs are proteins that bind to DNA-regulatory sequences (usually localized in the 5’-upstream region of target genes) to modulate the rate of gene transcription, which may result in increased or decreased gene transcription, protein synthesis, and subsequent altered cellular function. Research on inducing the differentiation of non-neuronal cells into neurons began as early as 20 years ago. It is worth noting that this process was inspired by the potential role of the various TFs. Heins et al. (2002) used retroviral vectors to overexpress the TF Pax6 in cultured astrocytes to generate neurons *in vitro*. Subsequently, they used another TF, Mash1, to transdifferentiate early postnatal astrocytes into neurons *in vitro* (Berninger et al., 2007). These data represent a crucial step in the identification of the molecular signals able to reinitiate the former neurogenic potential in astrocytes and may facilitate the use of the most abundant cell type in the mammalian CNS to replenish degenerating neurons. Based on this study, the researchers used additional neurogenic TFs, which resulted in a considerable improvement in the efficiency and maturity of induced neurons.

Moreover, other studies have used neural TFs of the bHLH (basic helix-loop-helix) family, including Achaete Scute Complex-like 1 (Ascl1), and Neuronal Differentiation 1 (NeuroD1), and Neurogenin2 (Ngn2), to promote the reprogramming of glia cells into neurons (Jorstad et al., 2017; Rivetti di Val Cervo et al., 2017). Torper et al. (2013) used viral delivery of the three neural conversion factors brain-2 (Brn2a), Ascl1, and myelin transcription factor-like 1 (Myt1l) to show that endogenous mouse astrocytes can be directly converted into neurons *in situ*. Then, Kempf et al. (2021) reprogrammed spinal cord astrocytes into mature neurons using the proneural factors Ascl1 and Ngn2. They also transformed mouse cortical astrocytes into pyramidal neurons by expressing the neural transcription factor Ngn2 alone and found that glial cells in different layers of the cerebral cortex could be directed to transform into neurons in the same layer (Mattugini et al., 2019).

Furthermore, Guo et al. (2014) showed that reactive glial cells in the cortex of stab-injured or Alzheimer’s disease (AD) model mice could be directly reprogrammed into functional neurons *in vivo* using retroviral expression of a single neural transcription factor, NeuroD1. NeuroD1 is a neural differentiation factor that is expressed in NSCs in the early stages of brain development and can make NSCs differentiate into neurons (Pang et al., 2011). By overexpressing NeuroD1, astrocytes can be reprogrammed into glutamatergic neurons, while NG2 cells can be reprogrammed into glutamatergic and GABAergic neurons (Guo et al., 2014). Based on this characteristic of NeuroD1, they found that when NeuroD1 was expressed in astrocytes, it could be transformed into neurons within a few weeks. So far, this trans-differentiation strategy has been promoted in stroke, AD, Parkinson’s disease (PD), Huntington’s disease, and other neurological diseases to increase neuronal regeneration and improve disease symptoms (Puls et al., 2020; Wu et al., 2020; Zhang et al., 2020), suggesting that NeuroD1 is a potential therapeutic target for future clinical treatment of massive neuronal loss.

In addition to overexpressing the transcription factor NeuroD1 in astrocytes, previous studies demonstrated that down-regulation of polypyrimidine tract binding protein 1 (PTBP1) expression in astrocytes can also induce conversion into neurons *in situ.* At present, neurogenesis gene therapy based on PTBP1 knockdown has achieved remarkable results and has been applied to various neurological disease models, such as stroke, Huntington’s disease, PD, and retinal damage (Qian et al., 2020; Wu et al., 2020; Zhou et al., 2020). However, recent studies doubt on this conclusion, showing that Ptbp1 deletion does not induce trans-differentiation of astrocytes into neurons in adult mouse retina and brain (Wang L. L. et al., 2021). Researchers used irreversibly labeled astrocytes with Sun1-GFP for lineage tracing. During their experiments, they did not observe any GFP-positive cells co-labeled with the neuronal markers NeuN and HuC/D in brain regions including cortex, striatum, and substantia nigra. Besides, Ptbp1 deletion did not make astrocytes trigger any neuron-like potentials, suggesting that their electrophysiological functions were not altered. In addition, by detecting the expression of glia-specific markers, researchers found that Ptbp1-deficient Müller glia retained expression of the glial marker SOX9 and did not detect gene expression characteristic of neurons, indicating that loss of Ptbp1 function did not significantly alter gene expression in Müller glia nor cause these cells to lose their identity (Hoang et al., 2021).

At present, the research on whether the deletion of Ptbp1 gene can lead to the trans-differentiation of astrocytes into neurons is still controversial, and some scholars have questioned that this false positive result is caused by a leaky neuronal expression of the GFAP-based AAV and transgene lines used to label astrocytes. But there is no doubt that it will become a research hotspot in the field of neural regeneration in the future, and provide new clinical treatment strategies for neurological disease.

### Astrocyte Reprogramming Through MicroRNAs

As discussed above, TFs regulate gene expression at the transcriptional level, while miRNAs, non-coding sequences of RNA, regulate gene expression at a posttranscriptional level, which mostly targets protein-coding mRNA transcripts via RNA silencing. miRNAs play an important role in cell differentiation, organ development, and cell fate determination, and are also involved in various human pathologies (Ha and Kim, 2014). Emerging studies have shown that many miRNAs are expressed in astrocytes, and some are involved in astrocyte differentiation, activation, and reprogramming activities (Bai et al., 2021). As miR-9 and miR-124 control multiple genes regulating neuronal differentiation and function, Yoo et al. (2011) proposed that these miRNAs might contribute to neuronal fates. Consequently, they converted fibroblasts into neurons with a single lentiviral vector that expressed both miR-124 and miR-9.

However, the research demonstrated that when using miRNA alone, the conversion success rate of fibroblasts was < 5%. To solve this problem, in subsequent experiments, the conversion efficiency was significantly increased to > 50% by mediating the expression of the neurogenic TF, NeuroD2 (Yoo et al., 2011). These studies demonstrate that the genetic circuit involving miRNAs may play a decisive role in the differentiation fate of neurons.

Furthermore, miRNAs contribute to astrocyte reprogramming by regulating miRNA targets. Mo et al. (2018) demonstrated that knockout of miR-365 can promote the transformation of astrocytes into neurons by up-regulating the 3’-UTR Pax6. Besides, miR-124 can promote neuronal differentiation by targeting polypyrimidine tract binding (PTB) protein and its homolog, neuronal PTB (nPTB) (Makeyev et al., 2007; Xue et al., 2013). PTB protein inhibits splicing of neuron-specific exons and is downregulated in the nervous system, whereas nPTB is up-regulated during neuronal differentiation. Thus, astrocytes could be directly reprogrammed into functional neurons *in vivo* through the mediation of the PTB/nPTB loop.

### Astrocyte Reprogramming Through Small Molecules

Astrocyte reprogramming through small molecules has some disadvantages, including complex operation and risk of tumor induction when inducing astrocyte reprogramming by viral infection. Therefore, the current clinical application is limited due to the consideration of delivery and safety. Small molecules are non-immunogenic and do not integrate with the genome, and their manipulation of intracellular targets is reversible. These features facilitate the ability of small molecules to determine the fate of cells *in vivo a*nd thus enable regenerating cells for tissue repair after CNS injury. Chen et al. previously used human cortical astrocytes in primary cultures for chemical reprogramming, they found that by gradually adding a mixture of nine small molecules (LDN193189, SB43154e2, TTNPB, Tzv, CHIR99021, VPA, DAPT, SAG, and Purmo), they were able to successfully reprogram human astrocytes into neurons that functioned normally. Small molecules act through two mechanisms in the successful trans-differentiation of this astrocytes into neurons: inhibit glial signaling pathways or activate neuronal signaling pathways. They successfully reprogrammed 67% of astrocytes into neurons within 8–10 days. Approximately 88% of these transdifferentiated neurons were glutamate neurons and 8% were GABAergic neurons, which could survive for > 5 months and form functional synaptic networks. Importantly, these newborn neurons survived for > 1 month when transplanted into the mouse brain and were integrated into the local neural circuitry of the host mouse (Zhang et al., 2015). In order to improve the efficiency of astrocytes reprogramming into neurons, researchers then examined the core-acting small molecules of these nine small molecules and found that the four core molecules (SLCD) including SB431542(S),LDN193189 (L), CHIR99021(C), and DAPT (D) appeared to be most critical in maintaining high reprogramming efficiency. They found that these four core small molecules achieve reprogramming of neurons mainly by regulating four signaling pathways including Notch, glycogen synthase kinase 3b (GSK-3b), transforming growth factor b (TGF-b), and bone morphogenetic protein (BMP) pathways. Such transdifferentiated neurons have normal function and can survive in cell culture for more than 7 months (Yin et al., 2019). This important discovery advances the research progress of astrocytes trans-differentiation and provides a potential treatment strategy for the current massive loss of neurons after stroke. In addition, Cheng et al. (2015) also confirmed that another combination of small molecule compounds (ISX-9, I-BET151, Valproic acid, Forskolin, Chir99021, Repsox) could induce the trans-differentiation of 70% of human astrocytes into neurons. The trans-differentiated neurons (including 70% glutamate and 7% cholinergic) survived for > 1 month when transplanted back into the mouse brain. Apart from that, Cheng et al. (2015) demonstrated that the chemical cocktail VCR (VPA, CHIR99021, Repsox) can directly convert astrocytes into neurons *in vitro* through activation of NeuroG2 and NeuroD1 expression independently of Notch signaling. These trans-differentiated neurons express neuronal markers such as DCX, NeuN, and Tuj1, and they are able to fire repetitive action potentials and spontaneously exhibit postsynaptic currents (Cheng et al., 2015).

However, astrocytes reprogrammed through small molecules *in vitro* need to be transplanted into the host, but high rates of xenograft rejection and low post-transplant survival limit their clinical application. Li et al. (2015) previously reported that a chemically defined cocktail, FICB (Forskolin, ISX9, CHIR99021, and I-BET151), could reprogram fibroblasts into functional neurons *in vitro*. Recently, they developed a chemical approach to convert endogenous astrocytes into neurons *in situ.* Such trans-differentiated neurons exhibited brain region-specific neuronal properties as well as electrophysiological characteristics of mature neurons. Besides, the trans-differentiated neurons can form direct afferent connectivity with surrounding neurons in the cortex (Ma et al., 2021).

The research results mentioned above show that the use of chemical small molecules can successfully achieve the trans-differentiation of astrocytes into neurons, thereby obtaining functional neurons. This provides a new therapeutic strategy for the massive loss of neurons after stroke, also provides prospects and directions for future research.

## Astrocyte Reprogramming Into Neurons in a Stroke Model

Stroke has high morbidity and mortality worldwide. Considerable neuronal death is a typical feature after stroke, causing irreversible damage to brain function. The death of neurons is impossible to reverse and neurons have a limited capacity to regenerate; thus, there is currently no effective treatment for stroke in the clinic. Therefore, replenishing the missing neurons in the injured area of the brain, and then reconstructing the functional connections between neurons is critical to promote post-stroke recovery. Current effective approaches include exogenous stem cell transplantation and endogenous stem cell mobilization. However, previous studies have shown that it is not only difficult for the transplanted cells to adapt to the new environment of the host but they are also easily attacked by endogenous immune cells after transplantation. On the other hand, endogenous stem cells in the brain are confined to only a few areas, and their numbers are limited. Therefore, there is an urgent need to further explore options for recovering large numbers of neurons after stroke. As astrocytes and neurons are derived from the same primitive progenitor cells, which are closer in cell developmental lineage, it is less difficult to convert between the two cell types. Furthermore, astrocytes are not only abundant in number but can also be replenished by self-splitting, which provides a good source cell type for converting into neurons. Therefore, many researchers have focused on astrocyte reprogramming into neurons during the past decade (Ge et al., 2020; Jiang et al., 2021).

After confirming that NeuroD1 can efficiently convert endogenous glial cells in the mouse brain into functional neurons *in situ*, Chen et al. (2020) transferred retrovirus carrying the NeuroD1 gene into the injury area of ischemic stroke to successfully reprogram astrocytes into neurons. Moreover, to provide a new therapeutic strategy for the clinic they used the gene therapy vector AAV to package and deliver NeuroD1 to ischemic lesions in the mouse brain and found that astrocytes overexpressing NeuroD1 could gradually be reprogrammed to functional neurons (Chen et al., 2020). It is worth noting that these transformed neurons could not only trigger repetitive action potentials but also establish functional synaptic connections with other neurons, indicating that these new neurons were integrated into neural circuits. Meanwhile, a series of behavioral experiments have also confirmed that after receiving NeuroD1-mediated gene therapy, the motor and cognitive functions of stroke animal models were also significantly improved (Chen et al., 2020). Recently, the same group further delivered the AAV-packaged NeuroD1 vector into astrocytes in the injured area in an ischemic stroke model of the primate macaque brain and successfully converted them into neurons (Ge et al., 2020). Additionally, they found that the microglia-mediated immune-inflammatory response was subsequently reduced in the macaque model, preventing further brain damage problems. In summary, this novel gene therapy mediated by NeuroD1 can produce new effective neurons in patients within 10–30 days of stroke, which may provide stroke patients with a wider treatment time window, thus solving an urgent clinical problem (Ge et al., 2020). Although further preclinical validation in primates is necessary, the success of *in situ* nerve regeneration in rhesus monkeys suggests that this gene therapy may offer a new alternative to stroke patients.

In addition to TF-induced astrocyte reprogramming, it has been reported that reactive astrocytes activated after stroke can indirectly differentiate into neurons. Magnusson et al. (2014) used YFP to label astrocytes in transgenic mice, and the results of immunofluorescence staining of brain tissue after MCAO showed that > 70% of YFP-labeled astrocytes could express the spiritual TF Ascl1 2 days after injury. After 2 weeks, Ascl1 + astrocytes aggregated to form a cluster of cells expressing the neuroblast marker DCX. Then, after 7 weeks, approximately 31% of DCX+ cell clusters differentiated into NeuN + neurons (Magnusson et al., 2014). Furthermore, Duan et al. (2015) reported that reactive astrocytes could undergo direct trans-differentiation to produce neurons in MCAO model mice. Three days after stroke, they found that only 47% of reactive astrocytes began expressing the NSC marker Nestin and the neuronal TF Pax6. While the immature neuron marker TUJ-1 was expressed on day 7 and NeuN was expressed on days 14 and 28. More than 90% of the transformed neurons had dopamine or glutamate receptors on their cell membranes, and these neurons had the same ability to synthesize and release neurotransmitters as neurons in normal brain tissue (Duan et al., 2015). These findings suggest that reactive astrocytes, which are activated in large numbers after stroke, represent a good source of neuronal regeneration. Although substantial progress has been made in this field so far, further mechanisms remain to be studied in the future.

## Opportunity of Astrocyte Reprogramming

The method of using astrocytes trans-differentiation into functional neurons after stroke has always been a research hotspot, which cannot only make up for the massive loss of neurons after stroke, but also provide a new strategy for clinical treatment. Astrocytes are morphologically and functionally activated and then become reactive astrocytes, which can quickly compensate for the loss of neurons. This process occurs *in vivo* without the many limitations faced by stem cell transplantation. With the rapid development of genetic lineage tracing, single-cell RNA transcriptome sequencing technology and AAV technology, the map of astrocyte reprogramming has become clearer. In the following, we will discuss potential opportunities in the future scientific research field in every aspect.

### Genetic Lineage Tracing Technology to Study Cell Fate Transition *in vivo*

Recently, genetic lineage tracing technology has gradually become a powerful tool for studying cell fate transitions *in vivo*. This technology is based on the Cre-loxP homologous recombinase system, which consists of two parts: (1) Cre recombinase expressed under the control of tissue or cell-specific promoters, and (2) a fluorescent protein (such as GFP) reporter gene (Kretzschmar and Watt, 2012). A loxP-STOP-loxP sequence is positioned in front of the reporter gene, which enables Cre recombinase to specifically recognize the loxP site and excise the STOP sequence in the middle, thereby activating the expression of the following reporter gene.

This specific excision is performed at the genomic level, which is permanent and irreversible, resulting in all Cre-expressing cells and their progeny being tagged with the reporter gene (Kretzschmar and Watt, 2012). At present, inducible genetic lineage tracing is more commonly used, in which Cre is fused with the human estrogen receptor (ER) to form a CreER fusion protein. Under normal circumstances, CreER binds to the heat shock protein HSP90 and stays in the cytoplasm, and cannot enter the nucleus. After induction with Tamoxifen, CreER can be released from HSP90 and enter the nucleus, and the Cre-loxP homologous recombination system reaction activates the expression of reporter genes and labels target cells (Tian et al., 2015). As inducible genetic lineage tracing technology is mainly controlled by tamoxifen, and given that the time and space factors are more flexible, this research method is favored by many researchers.

Furthermore, to investigate whether cardiomyocyte regeneration occurs after myocardial infarction, van Berlo et al. (2014) performed direct genetic lineage tracing of c-Kit+ stem cells using c-Kit-Cre and c-Kit MerCreMer (a cardiomyocyte-specific promoter) knock-in mice to track c-Kit + stem cells during adult cardiac physiological homeostasis and after cardiac injury. They demonstrated that endogenous adult cardiac c-Kit+ cells contributed very little (<0.008%) to cardiomyocyte regeneration and had no substantial significance for cardiac regeneration (van Berlo et al., 2014). However, the debate in this field is ongoing, and it remains to be determined whether the *in vivo* genetic tracking tools c-Kit-MerCreMer or c-Kit-CreER used in the above experiments have labeling efficiency problems. These *in vivo* tracking tools do not sufficiently label all c-Kit+ cardiac stem cells. Additionally, knock-in with the exogenous MerCreMer or CreER behind the promoter of the c-Kit locus results in the knockout of one allele of c-Kit, which may impair the differentiation of c-Kit cells into cardiomyocytes (Vicinanza et al., 2018).

Recently, researchers have continued to develop new tools to resolve this problem. He et al. (2019) used constitutive Cre to replace inducible CreER or aMHC-MerCreMer and inserted it after the last exon of the c-Kit gene, which not only achieved high-efficiency labeling but also retained the integrity and function of the c-Kit gene. They used this technique to demonstrate that c-Kit+ does not differentiate into cardiomyocytes during cardiac regeneration after cardiac injury repair (He et al., 2019). Additionally, due to the limited half-life of Tamoxifen, the inductive activity of Cre gradually decreases over time, leading to unsatisfactory long-term monitoring of proliferating cells. Therefore, He et al. (2021) developed another new technique called proliferation Tracer (ProTracer), which can detect cell proliferation continuously over an extended duration. They used two site-specific recombinases (Cre and Dre): R-DreER; Ki67-CrexER (Ki67-Cre-rox-ER-rox); R26–GFP to establish the ProTracer mice system. After induction by Tamoxifen, DreER can enter the nucleus, thereby converting CrexER into a constituent Cre element. Therefore, the formation of constitutive Ki67-Cre can be induced by Tamoxifen administration, and all proliferating cells can be labeled after administration to complete the continuous recording of proliferating cells (He et al., 2021).

The above studies on cardiomyocyte regeneration demonstrate that lineage tracing techniques must be carefully employed to draw accurate conclusions. Similarly, in the field of neuroscience, researchers should also practice more rigorous lineage tracing in the future research process. To better track the whole process of astrocytes reprogramming into neurons, researchers can consider using some proliferation markers such as Brdu and ki67. In addition, paying close attention to neurogenesis markers such as Doublecortin (DCX) is also an essential evidence in the process of trans-differentiation (Couillard-Despres et al., 2005). Through these indicators, researchers can rigorously and clearly track the fate changes of local astrocytes during the conversion process. Furthermore, previous study demonstrated that retroviral infection may activate microglia, leading to possible fusion with endogenous neurons, which may lead to false-positive results in experiments (Ackman et al., 2006). For the above reasons, rigorous lineage tracing is essential even for retrovirus-mediated fate transforming.

### Single-Cell RNA Transcriptome Sequencing Technology to Study Cell Fate Transition *in vivo*

Astrocyte reprogramming relies on precise and complex regulation, and although some progress has been made in this field, the detailed regulatory mechanism remains to be studied. Generally, most astrocytes are in a quiescent state. In the pathological process of disease or injury, astrocytes undergo a series of morphological and functional changes and thus become activated, following which, a few may transdifferentiate into neurons. Traditionally, the cell population is purified using surface markers. However, this cannot be used to distinguish transitional cells and highly heterogeneous cell populations, and it is difficult to realize the functional differences of a few cells through population analysis. Astrocyte reprogramming is a continuous process, during which astrocyte function declined, markers gradually decrease, and neuronal markers are gradually expressed. The molecular mechanisms and regulatory networks underlying this process are currently unclear. The development of single-cell sequencing techniques would address cellular heterogeneity at the individual cell level and improve understanding of astrocyte reprogramming processes.

Cebrian-Silla et al. (2021) presented a single-cell RNA-sequencing dataset of the adult mouse ventricular-subventricular zone (V-SVZ) revealing two populations of neural stem/progenitor cells (NSPCs) that reside in largely non-overlapping domains in either the dorsal or ventral V-SVZ. They also identified two subpopulations of young neurons that have gene expression profiles consistent with a dorsal or ventral origin (Cebrian-Silla et al., 2021). This study used novel markers to reveal the region-specific regulation of adult neurogenesis. This study also reminds us that newborn neurons do not necessarily express classical neuronal markers and may have their own unique gene expression signatures.

Recently, researchers have used “artificial DNA barcodes” as a lineage tracer, molecular marker, and single-cell sequencing technology as a tracer method to establish a dynamic map of gene expression in cell culture, zebrafish, and clawed frog early embryonic development, respectively (McKenna et al., 2016; Spanjaard et al., 2018). Sequencing data at intervals of minutes to hours has been combined to describe the characteristics of each cell, and then, based on the formation process of the embryo, a lineage roadmap was established for early embryonic development to form initial organs (Kester and van Oudenaarden, 2018). The sequencing-based lineage tracing methods are currently used to map clonal relationships between molecules and enable detailed comparisons between molecules and mitotic histories, which have been described and summarized in detail in the latest review (Wagner and Klein, 2020).

### Optimization Strategies for Adeno-Associated Viruses Vectors

Adeno-associated viruses (AAV) are common vectors for gene transmission and expression and have many advantages, including good safety, low immunogenicity, and stable expression. However, the infection efficiency of AAV is lower than that of retroviral vectors. By modifying and mutating the capsid of AAV, it is possible to screen out AAV mutants with high infection efficiency to facilitate the delivery of target genes by AAV vectors. Indeed, Mary et al. (2019) modified the capsid of AAV2 with glycosylation and increased the transgenic expression by 1.3–2.5-fold in three cell lines (HeLa, Huh7, and ARPE-19). This approach increased protein expression in the vitreous by approximately 2–4-fold and enhanced the overall retinal penetration *in vivo* experiments (Mary et al., 2019). Ran et al. (2020) conducted site-directed mutations of multiple surfaces exposed amino acid residues on raAV-DJ and RAAV-LK03 capsids. The results showed that the transduction efficiency of the mutant AAV vector was significantly higher than that of the wild type (Ran et al., 2020), suggesting that the site-directed mutation strategy is suitable for AAV modification and promoting the application of AAV in human gene therapy.

To deliver therapeutic genes more efficiently to specific tissues or cells, researchers need to further engineer AAV vectors to improve their targeting. Firstly, highly targeted AAV capsid structures can be screened by AAV-directed evolution. Davidsson et al. (2019) established the BRAVE (barcoded rational AAV vector evolution) method to optimize AAV. Using this method and clustering based on Hidden Markov Model (HMM) statistics, 25 synthetic capsid variants with refined properties were presented; these viruses were designed to target human dopamine neurons *in vivo*, and are transported along connected pathways in the brain, enabling unprecedented therapeutic accuracy (Davidsson et al., 2019).

The AAV promoter and serotype are two key factors that determine the dynamics of transgene expression. Specific tissues can be targeted by developing promoters suitable for specific cells or tissues. Nieuwenhuis et al. (2021) showed that AAV1 vectors with hCMV and sCAG promoters are highly expressed in neurons, astrocytes, and oligodendrocytes, and the mPGK promoter drives the expression in cortical neurons and oligodendrocytes. In contrast, AAV transduction with the hSYN promoter induced neuron-specific expression, including interneurons in peripheral neural networks and corticospinal neurons in layer V (Nieuwenhuis et al., 2021). This study demonstrated improved delivery of transgenes in the brain and spinal cord and provides a new viral tool for studying repair after spinal cord injury.

Notably, high doses of AAV can cause leakage into existing neurons and give false-positive results in experiments, in particular, with excessive amounts of AAV, such as titers of more than 1 × 10^13^ gc/mL. Puls et al. (2020). used titers of 1 × 10^8^ ∼ 1 × 10^10^ gc/mL to reduce false positives to ≤ 5%. Next, to efficiently regenerate many new neurons in the model of central nerve injury or disease, the concentration of AAV virus can be appropriately increased to 1 × 10^11^ ∼ 1 × 10^12^ gc/mL (Puls et al., 2020). This issue has been investigated by many scholars, and the issue of AAV titer should be further studied in the future.

## Challenge of Astrocyte Reprogramming

Most notable of these findings is the near-perfect “*in vivo* astrocyte-to-neuron reprogramming” achieved by the AAV vector overexpressing NeuroD1 or knocking down Ptbp1. These rapidly induced neurons show excellent potential, not only are their morphology, electrophysiological properties, and tissue arrangement nearly identical to those of endogenous neurons, but they also can repair glial scars, which has a significant therapeutic effect on stroke, PD, Huntington’s disease, and other diseases. However, many hurdles remain to be addressed in translating astrocyte reprogramming into the clinic.

In the most recent studies, scientists in the field of neural regeneration believe that the most critical question is whether these near-perfect neurons are derived from glial reprogramming. Wang L. L. et al. (2021) injected an AAV vector carrying both the reporter gene mCherry (a red fluorescent protein) and NeuroD1 into the mouse cerebral cortex and found that the reporter gene was mainly expressed in astrocytes on the fourth day after infection. At 17 days, the reporter gene in the control group was still concentrated in glial cells, while the reporter gene in the NeuroD1 group appeared to be mainly present in neurons, which were previously identified as “new neurons.” However, they did not observe an intermediate state in the trans-differentiation of glial cells into neurons, such as that observed in DCX-labeled immature neurons between days 4 and 17 after viral vector injection. Additionally, they applied a traumatic brain injury model to induce local glial activation and proliferation and used BrdU to label proliferative glial cells; however, the neuronal cells in the NeuroD1 group reporter gene were not derived from injury-activated glial cells (Wang L. L. et al., 2021). Next, they crossed Aldh1l1-CreERT2 with R26R-YFP to generate a tamoxifen-inducible mouse strain, in which it was possible to trace brain astrocytes with YFP. This strain labeled approximately 95% of the astrocytes in the cerebral cortex. Although the NeuroD1 group reporter gene mCherry was still observed in neurons in these mice, they did not carry YFP signals, indicating that they did not originate from brain glial cells. Additionally, they specifically labeled some motor neurons by injecting retroAAV carrying GFP into the spinal cord of mice, allowing it to enter the cerebral cortex through axons. This strategy enables neurons to express the GFP reporter gene and eliminates any other glial cell labeling in the brain, ensuring experimental labeling specificity. Surprisingly, they found many mCherry-positive neurons mediated by the NeuroD1 mediator, consistent with GFP-labeled endogenous neurons. This result strongly demonstrates that the so-called “new neurons” mediated by NeuroD1 are derived from certain endogenous neurons (Wang L. L. et al., 2021).

Similarly, Rao et al. (2021) used fluorescently labeled lineage tracer mice to confirm that microglia overexpressing DB could not be transformed into neurons. They proposed the following three basic principles needed to fully demonstrate glial-neuron reprogramming (Rao et al., 2021): (1) control groups should be well-designed with rigorous unambiguous lineage tracing control, excluding the possibility of viral leakage; (2) the glia-neuron transition process should be observed via unambiguous *in vivo*/*live* cell imaging evidence; (3) factor-mediated glia-neuronal trans-differentiation should be absent if this type of glial cell is cleared by plasma cells.

In this review, we put forward several suggestions with reference to the above-mentioned mainstream reprogramming strategies of astrocytes, hoping to further verify the authenticity of astrocyte reprogramming in subsequent studies.

## Conclusion and Perspective

The reason for the high morbidity and mortality of stroke in the world is closely related to the irreversible damage of neurons. There is currently lack of effective clinical treatment strategies for neuronal regeneration. In recent years, researchers have been working on the field of neurogenesis to solve this difficult problem. The use of astrocyte reprogramming to obtain functional neurons seems to be gradually gaining attention from researchers, which can solve the problem of massive neuronal death after stroke and neurodegenerative diseases (Ge et al., 2020). Reprogramming technology has gradually expanded from basic research to preclinical application and it may become an emerging treatment option for neurological diseases in the future. Compared to stem cell transplantation, the use of endogenous astrocytes to reprogram into newborn neurons is an economical and convenient strategy. It does not require the establishment of expensive stem cell banks and it avoids the long-term steps of stem cell cryopreservation and recovery, as well as the heterogeneity between different cell batches. Moreover, it does not have problems caused by foreign cell transplantation, including the risk of immune rejection and tumorigenesis.

However, this field also currently faces many challenges. Firstly, the success of astrocyte reprogramming is strongly related to astrocyte heterogeneity. As the morphology, function, and gene expression profiles of astrocytes from different brain regions are inconsistent, their reprogrammed products are also very different. Besides, some studies have reported that the conversion efficiency of glial cells into neurons is very different in gray and white matter regions; the conversion efficiency of astrocytes in gray matter is high, while that of astrocytes in white matter is low (Mattugini et al., 2019). Additionally, because of the altered state of astrocytes following nerve injury or degenerative disease, reactive astrocytes are highly efficient at reprogramming, whereas quiescent astrocytes are less efficient at transformation. Secondly, previous study stated that knocking down Ptbp1 readily converted glial cells into neurons in young mice but was difficult to achieve in older mice (Qian et al., 2020). On the contrary, the study recently posted in bioRxiv website showing that Ptbp1 deletion does not induce trans-differentiation of astrocytes into neurons in adult mouse retina and brain (Hoang et al., 2021). Controversies in this field have not yet been definitively resolved, suggesting that we should use more rigorous genetic tools in the future to validate the report on the glial-to-neuron reprogramming. Thirdly, AAV is currently the most commonly used vector for gene transmission and expression. However, high doses of AAV may present a risk of leakage, resulting in false positive results in experiments. The above-mentioned aspects indicate that there is still much work to be done in the future to achieve a truly rigorous reprogramming of astrocytes.

In recent years, the rapid development of lineage tracing and single-cell sequencing has made the map of astrocytes reprogramming clearer. The use of astrocyte reprogramming to supplement a large number of missing neurons after stroke and rebuild the functional connections between neurons provides a new strategy for the future clinical treatment after stroke and indicates a new direction in the future research field.

## Author Contributions

ZP and HL contributed equally to the review and were responsible for reviewing the literature and writing the original draft of the manuscript. QY was responsible for the investigation and data management. QX directed the revision of the manuscript. All authors contributed to the article and approved the submitted version.

## Conflict of Interest

The authors declare that the research was conducted in the absence of any commercial or financial relationships that could be construed as a potential conflict of interest.

## Publisher’s Note

All claims expressed in this article are solely those of the authors and do not necessarily represent those of their affiliated organizations, or those of the publisher, the editors and the reviewers. Any product that may be evaluated in this article, or claim that may be made by its manufacturer, is not guaranteed or endorsed by the publisher.
